# On Telegraphists’ Cramp

**Published:** 1878-08

**Authors:** 


### On Telegraphists' Cramp.

This affection is described by Dr. Onimus, in the *Gazette des Hopitaux.*

It is chiefly observed in those engaged in the manipulation of Morse's machine, and seems to arise from the difficulty of coordinating the motions which are required alternately for the formation of the dots and dashes. Much depends upon individual temperament and the condition of the nervous system, as the existence of more or less irritability seems quite as necessary for the production of this cramp as the frequent repetition of the same movements. Some employees who are naturally nervous and excitable have the cramp after only a short time of service, their general health suffering at the same time. The same circumstances operate in writers' cramp, this especially occurring when a great number of letters or despatches have to be executed in a given time, under a state of feverish activity. The direction of the movements also exerts an influence. An employee successively employed the thumb, the index, and the median finger, each of these manipulating during two or three months, but one after the other then being seized with the cramp. He then used his wrist, which after a while also refused service. As the expeditionary despatches are manipulated by a movement of the entire hand as well as of the fingers from above downward, when these vertical movements had become difficult an employee contrived a means of acting on the lever in a horizontal direction, by means of a thread stretched from a point of support to the lever. For a while he was able to forward his despatches, but these new movements soon became embarassed. and gave rise to the cramps.

The symptoms of the affection are more easily and more rapidly produced in women. The general symptons consist principally in palpitations, vertigo, sleeplessness, perhaps impairment of vision (most of the older and laborious employees wearing spectacles), and a sense of constriction opposite the nape of the neck, seeming to hold the back part of the head in a vice. This sensation is not uncommon in men of business rendered ill or over excited in important transactions in commerce, and it is especially met with when attempts are made to force intellectual functions that are already fatigued. To a state of over-excitement succeeds one of depression and melancholy,and moral and physical atony. Memory becomes bad, and, according to some, insanity may, in the course of some years, supervene on this pathological condition. During the progress of this pathological state the transmission of despatches presents some curious peculiarities, dependent on reflex movements produced by habit and in an unconscious manner. The hand does not always obey the determinations of the will; a word badly read is often correctly transmitted. On the other hand, an employee whose mode of transmission is naturally slow is not always, when dozing, interrupted in transmitting to his correspondent the ideas accompanying his half-dreamy state, for he continues to act on the lever and expedite his despatches. In some cases there exists, too, a state of things quite the opposite of spasm and rigidity, for the hand proceeds more rapidly than the will, and performs a series of movements which are coordinated and decipherable, but too rapid. It is especially after the manipulation has lasted for some time that these phenomena may be produced. Normally, it is only after an hour of work that the manipulation attains its maximum of rapidity.-*Medical and Surgical Reporter.*

